# Fatal Mistake

**Published:** 1869-03

**Authors:** 


Fatal Mistake.—Dr. W. M. Jennings, a prominent physician of Titusville, Pa., accidentally poisoned himself February 16th, and died in great agony. It appears that Dr. Jennings had been complaining of a pain in the chest, and finally visited a drug store to obtain medicine to relieve it. Arriving at the store, he inadvertently took a dose of what he supposed to be tincture of orange peel, but which unfortunately proved to be tincture of aconite. Powerful emetics and strong antidotes were administered, but all to no purpose. Vomiting was succeeded by convulsions, and a few hours after taking the poison he expired.—*Medical & Surgical Reporter.*

